# A Small Regulatory RNA Generated from the *malK* 5′ Untranslated Region Targets Gluconeogenesis in *Vibrio* Species

**DOI:** 10.1128/mSphere.00134-21

**Published:** 2021-06-30

**Authors:** Xing Luo, Marick Esberard, Philippe Bouloc, Annick Jacq

**Affiliations:** a Université Paris-Saclay, CEA, CNRS, Institute for Integrative Biology of the Cell (I2BC), Gif-sur-Yvette, France; University of Rochester

**Keywords:** *Vibrio*, sRNA, 5′ UTR, maltose regulon, gluconeogenesis

## Abstract

Vsr217 is a small RNA from Vibrio tasmaniensis LGP32, a pathogen associated with mortality events affecting juvenile oysters. The *vsr217* gene is located within the 5′ untranslated region (UTR) of *malK*, encoding the ATPase component of the maltose importer, and is conserved within the genus *Vibrio*. In the presence of maltose, *vsr217* is regulated by MalT, the positive regulator of the maltose regulon. *vsr217* is required in *cis* for the full expression of *malK*. In addition, Vsr217 acts in *trans* to downregulate the expression of *fbp* encoding fructose-1,6-bisphosphatase, an enzyme involved in gluconeogenesis. Thus, in the presence of maltose, the induction of Vsr217 is expected to promote glycolysis by negatively regulating the expression of a key enzyme of gluconeogenesis.

**IMPORTANCE** Juvenile pacific oysters have been subject in recent years to summer mortality episodes with deep economic consequences. The pathogen Vibrio tasmaniensis has been associated with such mortality events. For bacterial pathogens, survival within the host requires profound metabolic adaptations according to available resources. All kinds of regulatory elements, including noncoding RNAs, orchestrate this response. Oysters are rich in glycogen, a precursor of maltose, and we previously reported that *V. tasmaniensis* maltose-regulated genes are strongly induced during oyster infection. Here, we report the dual mechanism by which a small regulatory RNA, generated from the 5′ untranslated region of a gene belonging to the maltose regulon, acts both in *cis* and *trans*. In *cis*, it stimulates growth on maltose, and in *trans*, it downregulates the expression of a gene associated with gluconeogenesis, thus coordinating maltose utilization with central carbon metabolism.

## INTRODUCTION

In the last 20 years, the importance of noncoding RNAs (sRNAs) in bacterial regulatory networks has become widely recognized. The development of genome-wide transcriptomic studies has led to the exploration of transcriptomic landscapes of nonmodel bacterial organisms, either pathogens or those of environmental interest, leading to the discovery of many sRNAs (for reviews, see references 1–3). While classical bona fide sRNAs are expressed from intergenic regions, a newly recognized class of sRNAs includes sRNAs generated from 5′ and 3′ untranslated regions (UTRs) of coding genes that act in *trans* on genetically unlinked targets. A genome-wide search of small regulatory RNAs in Escherichia coli led to the identification of several sRNAs expressed from 5′ or 3′ UTRs (4), but only a few of them, mostly stemming from 3′ UTRs, have been characterized. For instance, CpxQ is generated from the 3′ UTR of *cpxP* mRNA, which encodes a periplasmic chaperone involved in the inner membrane stress response. CpxQ was shown to repress several inner membrane proteins, thus mitigating membrane stress (5). Another example is MicL, which is transcribed from a σ^E^-dependent promoter internal to the upstream protein coding sequence, and then cleaved to a shorter product to act in *cis* to regulate the expression of the major outer membrane lipoprotein Lpp (6). A few examples are associated with *Vibrio* species. In Vibrio cholerae, MicX is generated by an internal promoter within the *malG* open reading frame. The transcript is processed, and the shorter version, more stable and corresponding largely to the *malG* 3′ UTR, regulates an outer membrane protein, ChiP, annotated as a chitoporin, and an ABC transporter (7). Recently, two V. cholerae sRNAs, CarZ and OppZ, generated by processing of the 3’UTRs of *carAB* and *oppABCDF* mRNA, respectively, were shown to negatively regulate their respective operons (8).

*trans*-acting sRNAs can also be generated from 5′ UTRs. In the human pathogen Listeria monocytogenes, PrfA is a virulence regulator whose expression is controlled at the translational level by a thermosensor located in the 5′ UTR of the *prfA* transcript (9). However, two *S*-adenosylmethionine (SAM) riboswitches, SreA and SreB, not only regulate their downstream gene expression in *cis*, but also act in *trans* to target the 5′ UTR of the *prfA* mRNA (10). Another recent example is the sRNA rnTrpL (11) in Sinorhizobium meliloti, which is derived from a transcriptional attenuator of one of the three tryptophan operons and regulate in *trans* a second tryptophan operon, which does not possess the leader peptide-encoding attenuator. Other examples include Teg49 in Staphylococcus aureus, derived from the 5′ UTR of the virulence regulator gene *sarA* (12), or *malS* 5′ UTR, a Salmonella enterica noncoding RNA generated from the 5′ UTR of *malS* that was proposed to regulate in *trans hisG*, a gene involved in the histidine biosynthetic pathway (13).

Vibrio tasmaniensis LGP32 (previously V. splendidus LGP32) is a member of the V. splendidus clade (14) that has been associated with summer mortalities of juvenile Pacific oysters Crassostrea gigas (15). It is a facultative intracellular pathogen of oysters, invading the oyster hemocytes, the host immune cells (16–18). A genome-wide search for noncoding sRNAs in *V. tasmaniensis* LGP32 by transcriptome sequencing (RNA-seq) led us to identify 250 potential sRNAs, among which, several were confirmed by Northern blots and reverse transcription-PCRs (RT-PCRs) (19). More recently, a transcriptomic analysis of LGP32 within the host revealed that *vsr217* was one of the most highly induced genes compared to that during growth under seawater conditions (18). Interestingly, *vsr217* is located upstream of *malK*, a gene encoding the ATPase subunit of the maltose transporter, a member of the ABC transporter family (reviewed in reference 20). In addition, the maltose regulon was highly induced in oysters, a metabolic switch that can be related to the fact that oysters are rich in glycogen, a precursor of maltose (18).

Here, we show that *vsr217* is a member of the maltose regulon and is positively controlled by the transcriptional regulator MalT. While, within the 5′ UTR of *malK* mRNA, the Vsr217 sequence acts in *cis* to stabilize *malK* mRNA, it also generates an sRNA that acts in *trans* to repress the expression of a key gene involved in gluconeogenesis.

## RESULTS

### The genetic organization of *vsr217-malK* is conserved in different *Vibrio* species.

**FIG 1:**
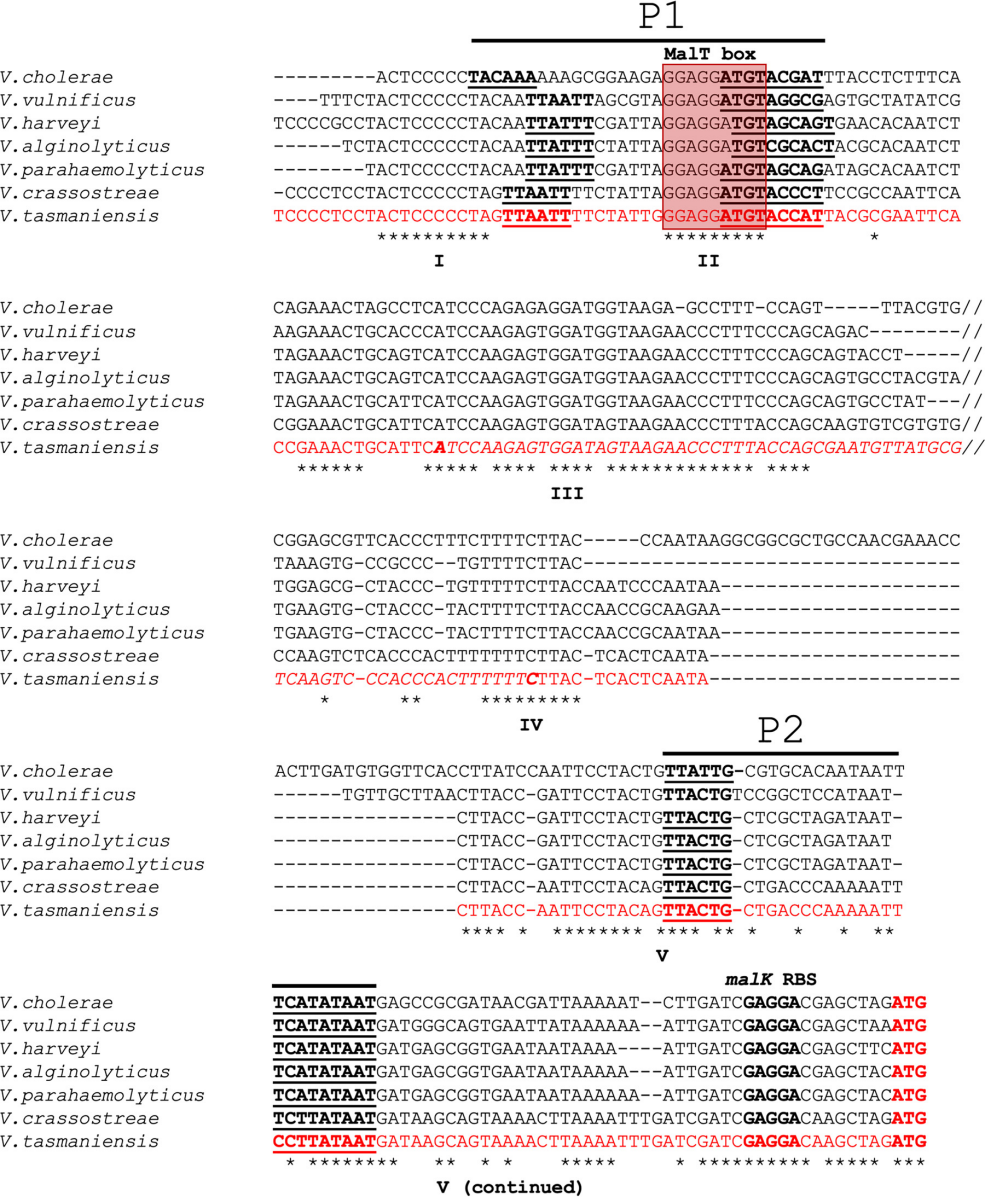
Vsr217 is conserved among *Vibrio* strains. The upstream regions of the *malK* gene of several *Vibrio* strains belonging to different clades (14) were aligned using Muscle (39). For reason of space, a less-conserved sequence between regions III and IV is not shown, as indicated by two slashes. Conserved nucleotides are indicated by * under the alignment. *V. tasmaniensis* sequence is in red, with the *vsr217* sequence in italic font. Important features are in boldface font, including Vsr217 5′ and 3′ ends; −35 and −10 regions of promoters predicted using BPROM (see Materials and Methods) are underlined. *malK* initiation codon is indicated in boldface red font.

Vsr217 is an sRNA from *V. tasmaniensis* expressed from the *malK* 5′ UTR. We investigated its conservation within the *Vibrio* genus by sequence comparison of *malK* upstream sequences from species representative of different *Vibrio* clades (14) (Fig. 1). Five regions are especially conserved. Region I is immediately upstream of a predicted −35 promoter sequence (P1). Region II overlaps with the −10 sequence of the P1 promoter and includes a putative MalT operator. MalT is the positive regulator of the maltose regulon in E. coli and other *Enterobacteriaceae* (21) which binds to MalT consensus sequences (22). Region III corresponds to the 5′ end of Vsr217 (see below) and includes the 32 first nucleotides of the sRNA. Region IV contains a conserved TTTTC motif present at the 3′ end of *vsr217*. Region V is the largest conserved sequence and contains a second predicted σ70 promoter (P2) as well as the ribosome binding site for *malK*. No Rho-independent terminator was predicted between the 3′ end of *vsr217* and *malK*. This analysis highlights the conservation of a large putative regulatory region upstream of *malK* in *Vibrio*.

### Vsr217 is induced by maltose in a MalT-dependent manner and is generated from the 5′ UTR of *malK*.

**FIG 2:**
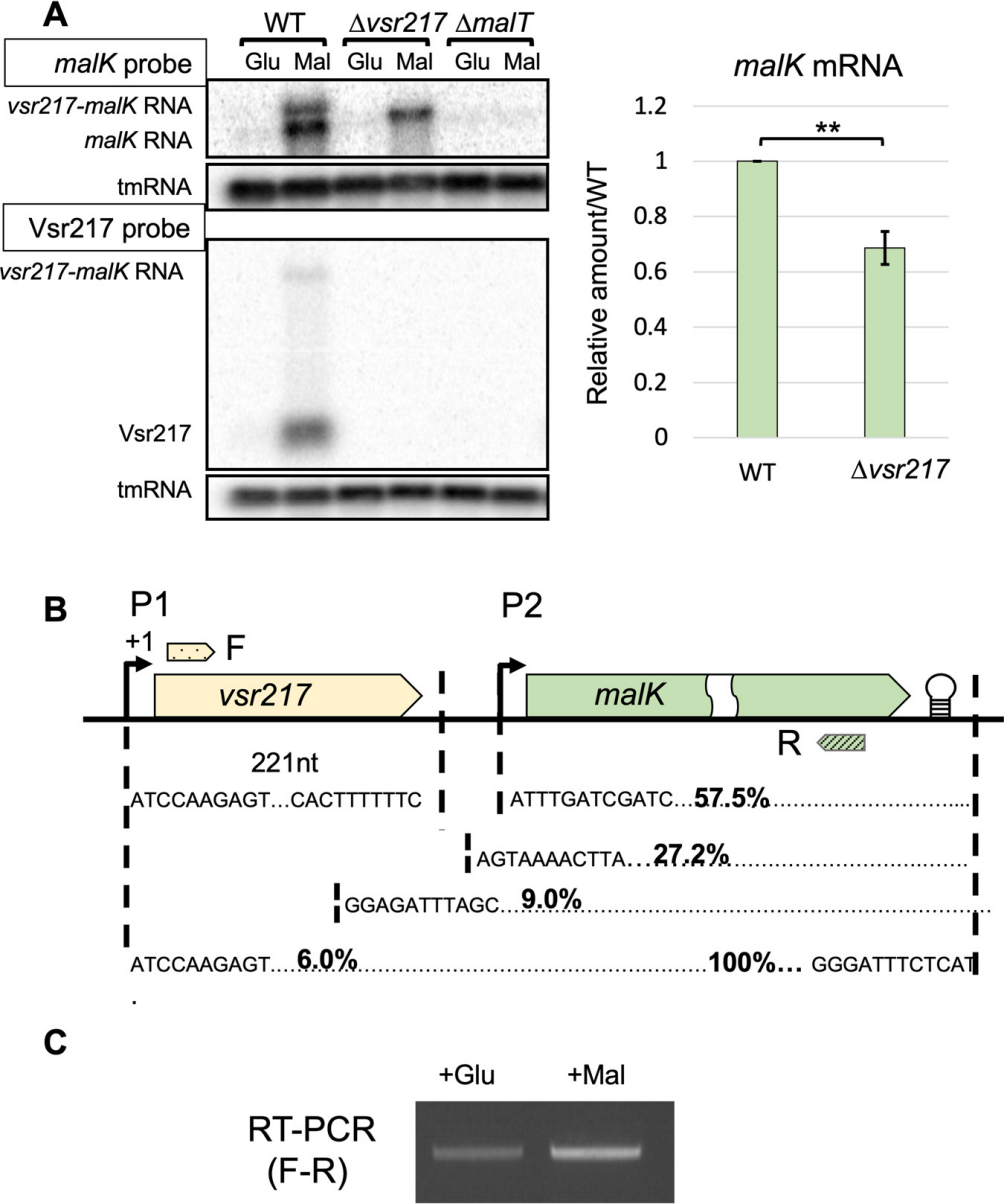
Vsr217 is induced by maltose in a MalT-dependent manner and is generated from the 5′ UTR of *malK*. Cells were grown in Zobell supplemented with glucose or maltose as indicated. (A, top left) Northern blots of *malK* in LGP32 WT, Δ*vsr217*, and Δ*malT* strains, using a *malK* probe. tmRNA was used for normalization. (Bottom left) Same membrane using a probe complementary to Vsr217. (A, right) Quantification of *malK* mRNA by ImageJ software. Error bars correspond to the standard errors of the means (*n* = 3). Significance was determined using a Student’s unpaired *t* test. **, *P* < 0.01. (B) Determination of 5′ and 3′ ends of Vsr217 and *malK* mRNA of cells grown in maltose. In each case, 20 clones were analyzed. The genetic organization is represented on top (not drawn to scale). Approximate position of primers used for RT-PCR is indicated (F and R). (C) Evidence for the existence of a Vsr217-*malK* transcript by RT-PCR using LGP32 WT total RNA extracted from cultures in either glucose or maltose as indicated.

The conserved localization of *vsr217* upstream of *malK* and the presence of a MalT box overlapping the putative P1 promoter of *vsr217* (Fig. 1) suggested that Vsr217 is a member of the maltose regulon controlled by MalT. The production of Vsr217 and *malK* mRNA was tested by Northern blots in the presence or absence of maltose (Fig. 2A). Both Vsr217 and *malK* mRNA were strongly induced by maltose, whereas no expression was detected in the presence of glucose. Vsr217 and *malK* mRNA were not detected in the *malT* mutant, indicating that their expression is MalT dependent. Under the maltose condition, a high-molecular-weight band was detected with both *vsr217* and *malK* probes, indicating the existence of a large transcript encompassing the two genes. A smaller band was detected with the *malK* probe but not with the *vsr217* probe, indicating the existence of a transcript including *malK* RNA without Vsr217. The transcription of *malK* was also tested in a mutant harboring a complete deletion of the *vsr217* sequence (see below and Fig. 2B). Using the *malK* probe, a single band was observed whose size was compatible with that of a *malK* transcript originating from the putative promoter P1, shortened by the absence of *vsr217* (Fig. 1A). Of note, *malK* mRNA quantity was reduced in the Δ*vsr217* mutant compared to that in its parental strain (Fig. 2A).

The 5′ and 3′ ends of transcripts from the *vsr217-malK* locus were determined by 5′ and 3′ rapid amplification of cDNA ends (RACE) mappings (Fig. 2B). For Vsr217, all clones displayed the same extremities. The Vsr217 5′ end was compatible with a transcription stemming from the P1 promoter, while the transcript terminated at the level of a conserved TTTTC motif. In contrast, for *malK* mRNA, if the 3′ end was unique, due to the presence of a Rho-independent terminator, different *malK* 5′ ends were identified. The majority of them (57.5%) corresponded to a transcription start site from the P2 promoter. Other 5′ end sequences mapped upstream of P2, some of them within *vsr217*. Among them, 6% had a 5′ end corresponding to a transcription initiation from P1, hence including the whole Vsr217 sequence (Fig. 2B). The existence of a *vsr217-malK* transcript was also confirmed by RT-PCR, whose amount increased in the presence of maltose (Fig. 2C).

We concluded that Vsr217 originates from the *malK* P1 promoter. The existence of transcripts encompassing Vsr217-*malK* mRNA suggested that Vsr217 is generated from *malK* 5′ UTR and that processing of the 5′ end of this larger transcript generates *malK* transcripts.

### Deletion of *vsr217* results in *malK* mRNA instability.

**FIG 3:**
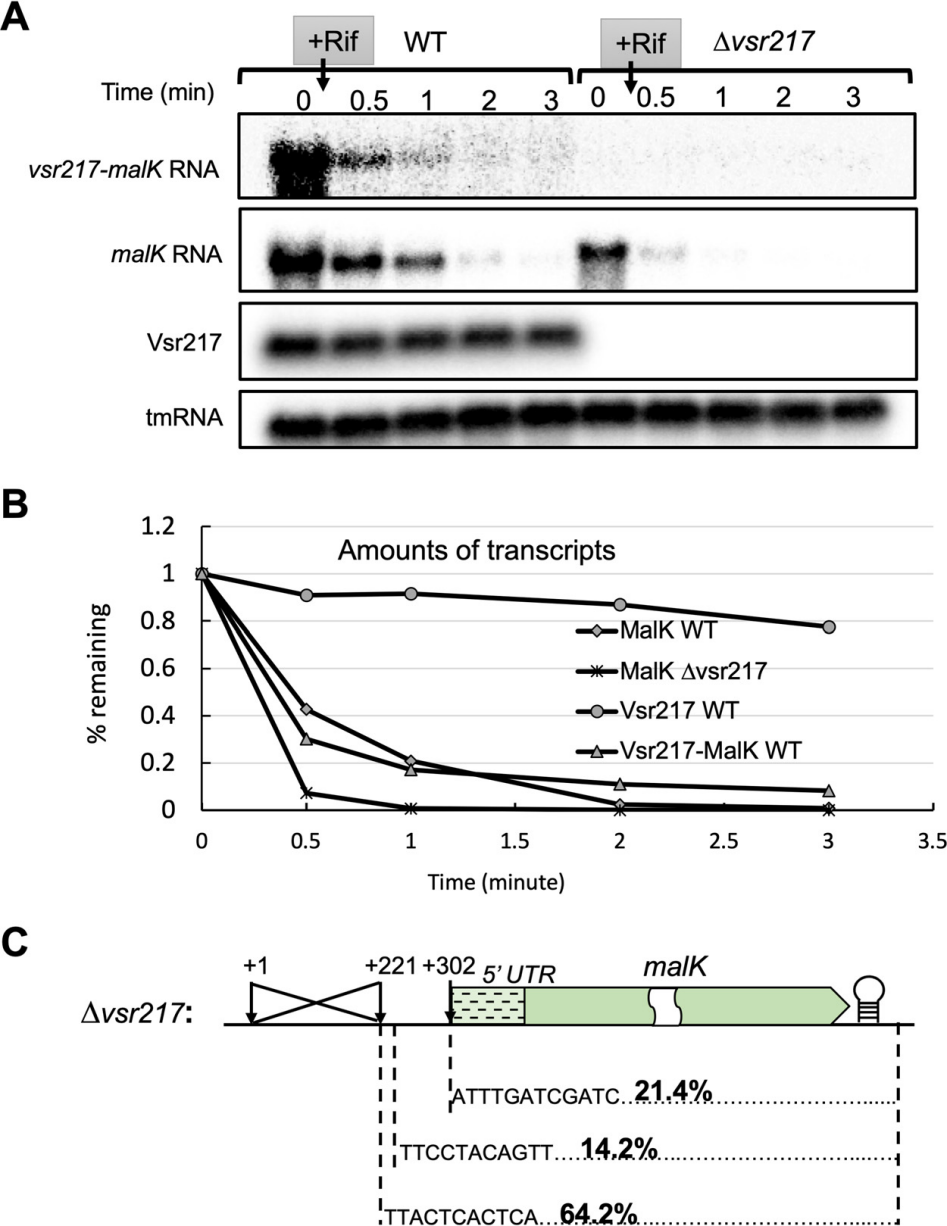
Deleting *vsr217* leads to an instability of *malK* mRNA. (A) Amounts of *malK* mRNA and Vsr217 from cells growing in minimal medium supplemented with maltose (OD_600_ ≈ 0.4) were determined by Northern blotting over time after adding rifampicin to block transcription. (B) Kinetics of degradation of *malK* mRNA and Vsr217 determined from the experiment presented in panel A. (C) 5′ ends of the *malK* mRNAs in the Δ*vsr217* mutant.

The amount of *malK* mRNA was reduced in the absence of *vsr217* (Fig. 2A), possibly because of *malK* mRNA destabilization. To check this, the half-life of Vsr217 and *malK* and *vsr217-malK* transcripts was determined in the wild-type (WT) and Δ*vsr217* strains (Fig. 3A and B). Vsr217 was a stable sRNA (half-life [*t*_1/2_], ∼7.5 min), whereas *malK* and *vsr217-malK* transcripts were unstable (*t*_1/2_, ∼30 s). Deletion of *vsr217* resulted in an even more unstable *malK* transcript (*t*_1/2_, ∼15 s).

The 5′ end of the *malK* mRNA in the Δ*vsr217* mutant was determined by 5′ RACE. In contrast to the parental strain, the majority of the *malK* transcripts (64.2%) in the Δ*vsr217* mutant had a 5′ end generated from the P1 promoter, whereas only 21.4% corresponded to transcripts generated from the P2 promoter (Fig. 3C).

Does the *malK* mRNA level reduction in the Δ*vsr217* mutant affect growth in the presence of maltose as the sole carbon source? Indeed, when grown in minimal medium supplemented with maltose, the Δ*vsr217* mutant had a lower growth rate than its parental strain. This difference was not observed in minimal medium supplemented with glucose (see Fig. S1 in the supplemental material). The maltose-dependent growth defect of the Δ*vsr217* mutant was corrected by the presence of a plasmid expressing *malK* only but not by a plasmid expressing *vsr217* under the P1 promoter (Table 1). We concluded that the growth defect in the mutant is due to the *cis* effect of the deletion of *vsr217* on *malK* mRNA stability, leading to the decrease of the message.

**TABLE 1:** Strains and plasmids used in this study

Strain or plasmid	Relevant characteristics	Reference or source
Strains		
*V. tasmaniensis*		
LGP32	Pathogen of oyster *C. gigas*	40
Δ*vsr217*	LGP32 carrying a deletion of *vsr217*	This study
Δ*malT*	LGP32 carrying a deletion of *malT*	This study
Δ*polyT*	LGP32 carrying a deletion of polyT at the 3′ end of *vsr217*	This study
E. coli		
DH5α	F^−^ *endA1 glnV44 thi-1 recA1 relA1 gyrA96 deoR nupG purB20 φ80dlacZ*ΔM15 Δ(*lacZYA-argF*)*U169 hsdR17* (r_K_^−^ m_K_^+^) λ^−^	Lab collection
MG1655	F^−^ λ^−^ *ilvG^−^ rfb-50 rph-1*	Lab collection
MG1655 Δ*hfq*	F^−^ λ^−^ *ilvG^−^ rfb-50 rph-1* Δ*hfq*	41
Π3813	E. coli K-12 *lacIQ thi1 supE44 endA1 recA1 hsdR17 gyrA462 zei298*::tn*10* [Tc] Δ*thyA*::(*erm-pir116*)[Er]; cloning host	42
GEB883	E. coli K-12 Δ*dapA*::*erm pir* [Er] *RP4-2* Δ*recA gyrA462 zei298*::Tn*10* [Tc]; donor strain for conjugation	34
Plasmids		
pSW7848	Suicide vector with an R6K origin, requiring the Pir protein for its replication, pBAD-ccdB, Cm^r^	35, 43
pSW7848-Δ*vsr217*	pSW7848 carrying the mutant allele Δ*vsr217*, Cm^r^	This study
pSW7848-Δ*malT*	pSW7848 carrying an in-frame deletion of *malK*, Cm^r^	This study
pSW7848-Δ*polyT*	pSW7848 carrying the polyT 13-nt deletion at the 3′ end of *vsr217*, Cm^r^	This study
pGEB12	Replicative, pSU18 with *orip15A*, *oriT*_RP4_, Cm^r^	44
pP1-P2-MalK	pGEB12, Cm^r^, *malK* under the control of P1-P2 promoters	This study
pP1-Vsr217	pGEB12, Cm^r^, *vsr217* under the control of *vsr217*-*malK* P1 promoter	This study
pZE12MCS	Based on pZE12-Luc, P_LlacO_, *ColE1 Ori1*, Amp^r^	26
pPlac-Vsr217	pZE12MCS, P_LlacO_-*vsr217*, Amp^r^	This study
pXG-10	pSC101* origin of replication (low copy), P_LtetO_ promoter, *gfp*^+^, Cm^r^	26
pPtet-Fbp-GFP	pXG-10 [−125, +3]-*fbp*::*gfp*, Cm^r^	This study
pXG-10Δalpha	pXG-10, Δalpha, Cm^r^	This study

10.1128/mSphere.00134-21.1 FIG S1 The Δ*vsr217* mutant has a growth defect in maltose as a carbon source, which is suppressed by MalK but not by Vsr217. (A) Growth of LGP32 WT and Δ*vsr217* strains in minimal medium supplemented by glucose or maltose. (B) Growth of WT and Δ*vsr217* strains in minimal medium supplemented by maltose in presence of plasmids expressing either Vsr217 (pP1-Vsr217, see Table 1) or MalK (pP1-P2-MalK). The empty plasmid pGEB12 was used as a control. Download FIG S1, PDF file, 0.1 MB. Copyright © 2021 Luo et al. 2021 Luo et al. https://creativecommons.org/licenses/by/4.0/ This content is distributed under the terms of the Creative Commons Attribution 4.0 International license.

### A U-rich terminal motif of Vsr217 is a stabilizing factor for Vsr217 but not for *malK* mRNA.

**FIG 4:**
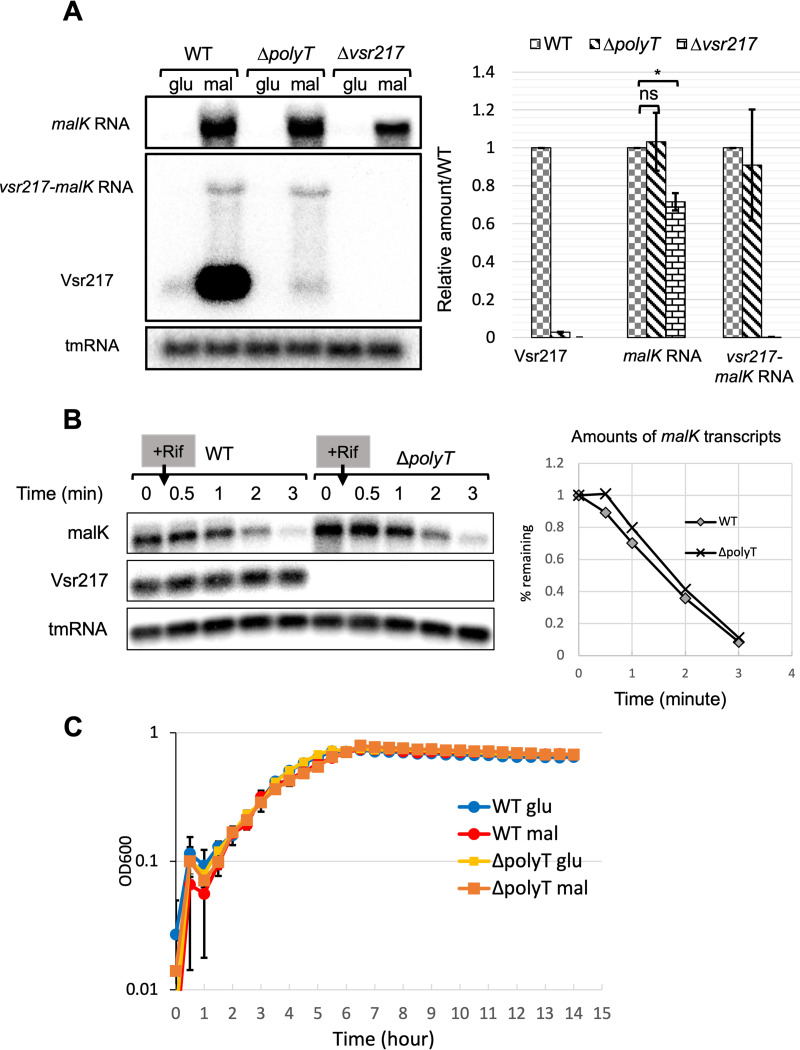
Role of the polyU motif at the 3′ end of *vsr217* in the expression of *vsr217* and *malK*. (A, left) Northern blots with probes for *malK* mRNA (top) and Vsr217 (bottom) in WT, Δ*polyT*, and *vsr217* mutant strains grown in minimal medium supplemented with glucose (glu) or maltose (mal). (Right) Northern blot RNA quantifications by ImageJ software. Results are the means from two experiments. Error bars correspond to the standard errors of the means. Significance was determined using a Student’s unpaired *t* test: *, *P* < 0.05. (B) Amounts of *malK* mRNA from cells growing in minimal medium supplemented with maltose (OD_600_ ≈ 0.4) were determined over time in the WT and Δ*polyT* mutant by Northern blotting after adding rifampicin to block transcription. (Right) Kinetics of degradation of *malK* mRNA determined from the experiment presented on the left. (C) Growth of the WT and Δ*polyT* strains in minimal medium supplemented with glucose or maltose.

Vsr217 contains a conserved U-rich motif at its 3′ end (Fig. 1A). We hypothesized that it could play a role in generating Vsr217, either by promoting transcription termination, by being a processing site, or by contributing to its stability. To test this, we constructed a *vsr217* mutant lacking its 13 last nucleotides (Δ*polyT*) and checked by Northern blotting how the deletion would affect the amounts of Vsr217, *malK* mRNA, and their precursor (Fig. 4A).

The absence of the last 13 nucleotides (Δ*polyT* mutant) led to a 100-fold reduction of Vsr217 compared to that in the WT strain, whereas the amount of *malK* mRNA was not affected in contrast to what was observed in the Δ*vsr217* mutant. Consistent with this result, we observed that *malK* mRNA stability did not decrease in the Δ*polyT* mutant compared to that in the WT strain, contrary to Vsr217 stability (Fig. 4B). Surprisingly, the quantity of the precursor transcript *vsr217-malK* mRNA was also not affected in the Δ*polyT* mutant as could have been expected if the deleted terminal motif was involved in a processing event generating Vsr217 and *malK* mRNA. Altogether, our results suggest that the U-rich terminal motif is involved in Vsr217 stability but not in *malK* mRNA stability.

In addition, no growth difference was observed between the Δ*polyT* mutant and its parental strain in minimal medium supplemented with either glucose or maltose (Fig. 4C), confirming that the growth defect observed in the Δ*vsr217* mutant was due to a *cis* effect on *malK* expression and not to the absence of Vsr217 production.

### Vsr217 affects the expression of *fbp*, a gluconeogenic enzyme.

The existence of a stable, abundant Vsr217 sRNA suggests a *trans*-acting function on targets possibly not directly related to maltose utilization but nevertheless regulated in response to maltose. Putative Vsr217 targets were searched by identifying proteins whose expression levels varied between the WT and Δ*vsr217* strains, especially when grown in maltose. Using a proteomic mass spectrometry approach, 2,104 proteins from 4,371 coding DNA sequences (CDSs) harbored by the *V. tasmaniensis* genome were identified. In glucose, the protein contents were the same between the two strains. However, in maltose, the amounts of 22 proteins were significantly altered (fold change of >2 or <0.5, *P* value < 0.05) in the mutant, with 11 increasing and 11 decreasing (Table 2). Among them, MalK decreased in the Δ*vsr217* mutant, consistent with the reduced amount of *malK* mRNA (Fig. 2).

**TABLE 2:** Proteins whose level is affected by Vsr217 in maltose

Locus tag	Fasta header	No. of peptides	Fold change for Δ*vsr217*/WT in Mal	*P* value	Fold change Δ*vsr217*/WT in Glu	*P* value	Rank CopraRNA	CopraRNA FDR^*a*^
Upregulated proteins								
VS_II0899	Prolyl aminopeptidase	10	6.81	0.00	0.88	0.89	>100	>0.23
VS_II0126	Oxidoreductase	8	5.84	0.05	1.00	NaN	>100	>0.23
VS_II0346	Glyceraldehyde-3-phosphate dehydrogenase GapC	18	3.33	0.01	1.11	0.81	>100	>0.23
VS_II0148	Catalase	10	3.03	0.04	1.00	NaN	>100	>0.23
VS_1054	Histidine transport ATP-binding protein HisP	4	3.02	0.03	1.00	NaN	>100	>0.23
VS_1129	Signal transduction histidine kinase	4	2.57	0.01	1.00	NaN	>100	>0.23
VS_II1090	Phosphomethylpyrimidine kinase	2	2.40	0.03	1.00	NaN	>100	>0.23
VS_2771	Fructose-1,6-bisphosphatase Fbp	17	2.36	0.00	1.16	0.18	8	0.101
VS_II1225	Phosphoenolpyruvate synthase PpsA	40	2.11	0.01	1.21	0.67	>100	>0.23
VS_2300	Arsenate reductase	3	2.09	0.02	0.97	0.97	>100	>0.23
VS_II0877	MoxR-like ATPase	6	2.01	0.03	1.00	NaN	>100	>0.23
Downregulated proteins								
VS_2926	Ornithine carbamoyltransferase	17	0.49	0.04	1.17	0.76	>100	>0.23
VS_2263	Glutathione-regulated potassium-efflux system protein kefb	4	0.48	0.03	1.07	0.83	>100	>0.23
VS_II0953	Nitrate reductase, large subunit	3	0.45	0.04	1.00	NaN	>100	>0.23
VS_2312	Arsenate reductase	4	0.45	0.04	1.00	NaN	>100	>0.23
VS_II0935	ABC transporter substrate-binding protein	7	0.43	0.04	1.42	0.35	>100	>0.23
VS_II0219	Maltose/maltodextrin transporter ATP-binding protein MalK	23	0.42	0.00	0.47	0.10	>100	>0.23
VS_1158	Hypothetical protein VS_1158	15	0.39	0.03	2.01	0.15	>100	>0.23
VS_II0405	TRAP dicarboxylate transporter subunit DctP	14	0.37	0.03	0.67	0.29	>100	>0.23
VS_2935	Sodium/solute symporter	3	0.23	0.04	1.00	NaN	>100	>0.23
VS_0474	Glutamate synthase (NADPH) large chain	64	0.19	0.00	1.02	0.39	>100	>0.23
VS_1157	Histidine kinase	8	0.13	0.00	0.75	0.71	>100	>0.23

a FDR, false-discovery rate.

Among these 22 potential targets of Vsr217, to identify candidates for direct mRNA targets, we used the software CopraRNA, which predicts sRNA targets and their pairing regions with the sRNA by comparative genomics (23). From the list of proteins whose levels were affected in the Δ*vsr217* mutant, only one came out in the list of the first 100 best candidates for direct interaction: VS_2771, ranked 8th by CopraRNA, corresponds to Fbp, a class 1 fructose-1,6-bisphosphatase that catalyzes the conversion of fructose-1,6-bisphosphate to fructose-6-phosphate, a rate-limiting step of the gluconeogenesis pathway (Table 2). Fbp expression increased in the Δ*vsr217* mutant, suggesting that Vsr217 could be a negative regulator of *fbp*.

**FIG 5:**
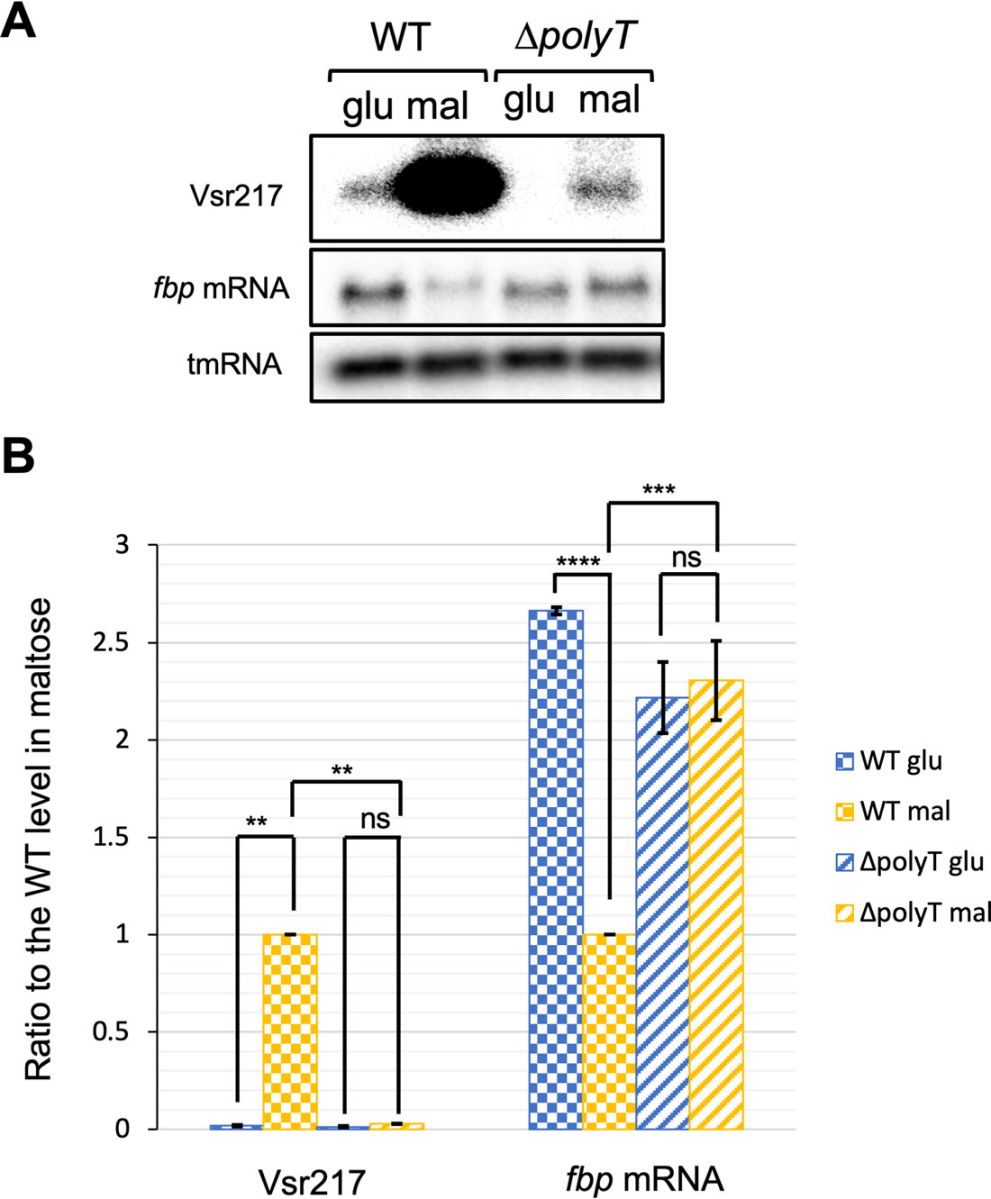
Decrease of Vsr217 results in an increase of *fbp* mRNA. (A) Detection of *fbp* mRNA in the WT strain and the Δ*polyT* mutant. (B) Quantification of Northern blot results using ImageJ software. Results are the means from two independent experiments, and error bars correspond to the standard errors of the means. The significance of observed differences was determined by two-way analysis of variance (ANOVA). ns, *P* > 0.05; **, *P* < 0.01; ***, *P* < 0.001; ****, *P* < 0.00001.

Since posttranscriptional inhibition by sRNAs often leads to a stability reduction of target mRNAs (24), we determined by Northern blotting the amount of *fbp* mRNA in minimal medium supplemented by either glucose or maltose (Fig. 5). To circumvent the *cis* effect of *vsr217* RNA on *malK* expression, we took advantage of the Δ*polyT* mutant, which has a drastically reduced amount of Vsr217 but still retains a wild-type expression level of *malK*. *fbp* was repressed in maltose compared to that in glucose in the WT, a difference that was abolished in the Δ*polyT* mutant, suggesting that Vsr217 downregulates *fbp* expression.

### *fbp* mRNA is a direct target of Vsr217.

**FIG 6:**
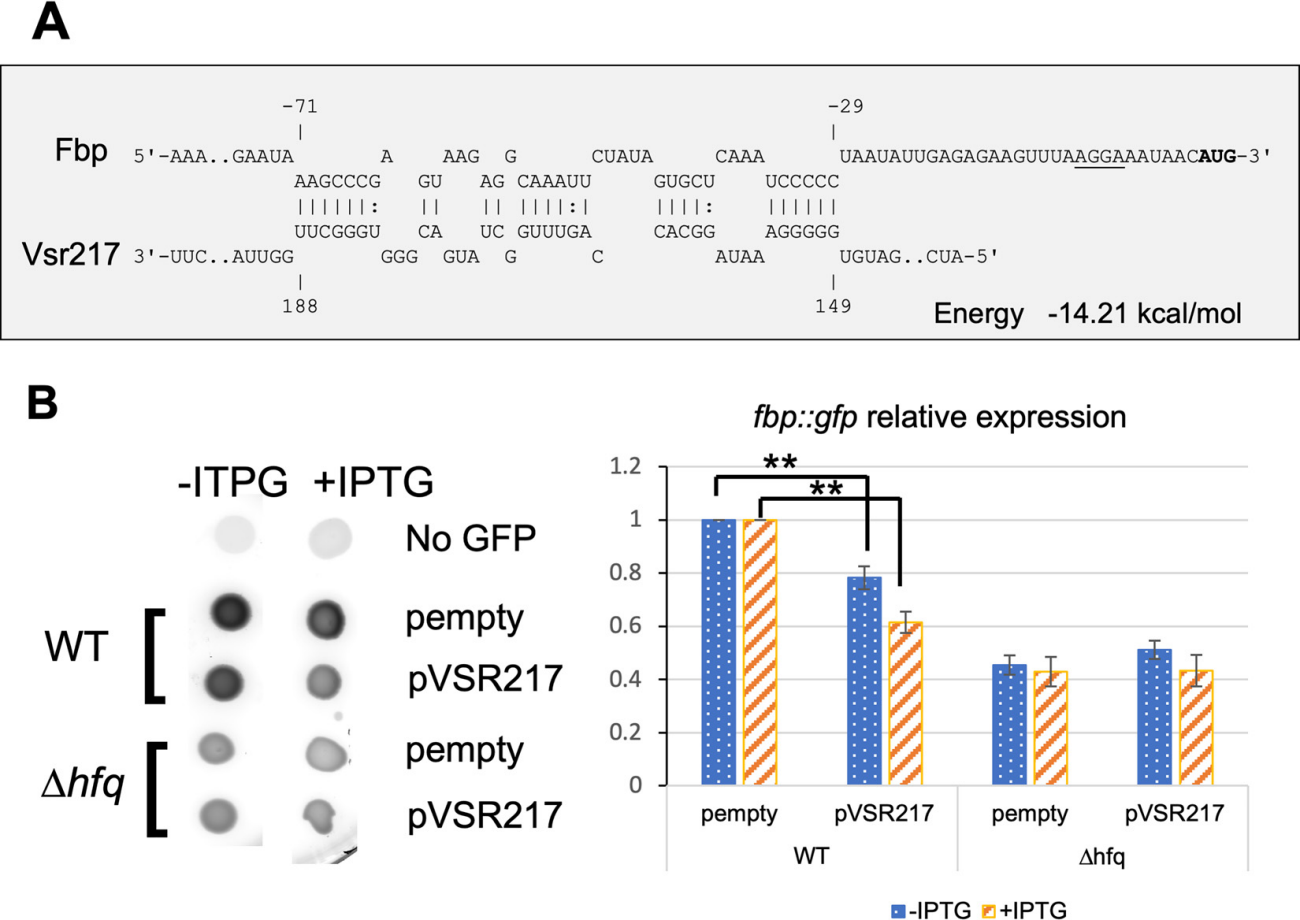
Posttranscriptional *fbp* mRNA downregulation by Vsr217. (A) Interaction between Vsr217 and *fbp* mRNA predicted by IntaRNA (23). (B) Effect of pPlac-Vsr217 on the expression of a translational fusion *fbp*::*gfp* including the putative interaction region between Vsr217 and *fbp* mRNA. Overnight (O/N) cultures were spotted onto a rich medium supplemented or not with IPTG. (Left) Following incubation, plates were scanned for fluorescence using a Typhoon imager. (Right) Fluorescence quantification by ImageJ software (*n* = 4). Values obtained for cells not producing GFP were deducted. Signal was then normalized to the level of the parental strain carrying the empty plasmid. Significance was determined by pairwise Student’s unpaired *t* tests. **, *P* < 0.01.

A biocomputing analysis using IntaRNA identified a potential base-pairing region between Vsr217 and *fbp* mRNA, ending 29 nucleotides (nt) upstream of the *fbp* initiation codon (Fig. 6A). To confirm a putative direct interaction, the Vsr217 *trans* activity on *fbp* expression was tested in E. coli using a two-plasmid-based reporter system (25, 26). The first plasmid carried the *vsr217* gene cloned under a P_L_*_lacO_* promoter (pPLac-Vsr217). The second plasmid contained an in-frame gene fusion between the predicted pairing region of *fbp* mRNA including *fbp* initiation codon and the second codon of the *gfp* CDS (Table 1). The reporter fusion was cloned under the control of a P_Ltet_ promoter, which is constitutive in the absence of a TetR repressor. pPtet-Fbp-GFP was introduced in E. coli together with pPlac-Vsr217, and the effect of Vsr217 production on the expression of *fbp* was monitored via fluorescence measurements (see Materials and Methods).

The presence of pPlac-Vsr217 plasmid led to a 20% reduction in the expression of the *fbp-gfp* fusion compared to that in the strain containing a control empty plasmid (Fig. 6B). Expression was further reduced to ∼40% when isopropyl-β-d-thiogalactopyranoside (IPTG) was added to fully induce *vsr217* expression.

The RNA chaperone Hfq is often required for the interaction of sRNAs with their targets, including in *Vibrio* (27–29). To determine whether the potential interaction between Vsr217 and *fbp* mRNA was dependent upon Hfq, we also transformed a MG1655 Δ*hfq* mutant with the same set of plasmids.

The *fbp-gfp* fusion had a 2-fold reduction of expression in the Δ*hfq* mutant compared to that in the WT strain, but the presence of pPlac-Vsr217 had no effect in the absence of Hfq, indicating that the interaction was Hfq dependent. Altogether, these results indicate that *fbp* mRNA is submitted to posttranscriptional inhibition by Vsr217 in an Hfq-dependent manner.

## DISCUSSION

*V. tasmaniensis* LGP32 Vsr217, identified in a genome-wide search for sRNAs (19), is highly induced during oyster infection (18). In this study, we show that *vsr217* is a conserved gene in *Vibrio*, located upstream of *malK*, which is inducible by maltose in a MalT-dependent manner. Induction of Vsr217 by maltose could account for its high expression in oysters. Indeed, the latter are rich in glycogen, a precursor of maltose, and the maltose regulon of *V. tasmaniensis* is induced upon oyster colonization (18).

An original feature of Vsr217 is that it is generated from the 5′ UTR of *malK* mRNA as attested by the detection of a *vsr217-malK* RNA. *malK* is transcribed from two promoters, P1, upstream of *vsr217*, and P2, located in the intergenic region between *vsr217* and *malK*. P2 is responsible for the transcription of more than half of *malK* transcripts. The other half was distributed between transcripts with several 5′ ends that could correspond to the results of processing of transcripts stemming from the P1 promoter. Altogether, these results are in favor of Vsr217 being generated by the processing of the larger transcript stemming from the P1 promoter, although we cannot exclude that Vsr217, which is much more stable than the *malK* transcript(s), is generated by termination of transcription at its unique 3′ end. However, no Rho-independent terminator was detected to support this hypothesis. In addition, we found that deleting the conserved 13 last nucleotides of *vsr217* (called here polyT motif) led to a nearly complete absence of Vsr217 but had no effect on *vsr217-malK* or *malK* mRNA levels. These results suggest that the polyT motif is important for Vsr217 stability but not for *vsr217-malK* processing.

Vsr217 RNA has two functions. In *cis*, it affects the stability of the *malK* mRNA, which decreases in the Δ*vsr217* deletion strain, leading to a reduction of MalK and a decreased ability to use maltose as a carbon source, which is suppressed by the expression in *trans* of *malK* but not of *vsr217*. In addition, Vsr217 acts as a bona fide sRNA to control the expression of *fbp*, a gene involved in the neoglucogenesis/glycolytic pathway. A decrease of *fbp* mRNA was observed in the presence of maltose, when Vsr217 is strongly induced, whereas no change was observed in glucose. This decrease was abolished in the Δ*polyT* mutant, confirming that it is Vsr217 dependent in the WT strain. Direct interaction between Vsr217 and *fbp* was further supported by Vsr217-dependent downregulation of a *fbp-gfp* reporter fusion in E. coli (Fig. 6). In addition, we found that the Vsr217-dependent posttranscriptional downregulation of the *fbp-gfp* fusion is indeed dependent upon Hfq in E. coli. *V. tasmaniensis* Hfq has 94% identity and 99% similarity with E. coli Hfq. Accordingly, it is likely that the Vsr217-*fbp* interaction is dependent upon Hfq in *V. tasmaniensis* as well.

Interestingly, *fbp* encodes one of the two enzymes in the glycolytic/gluconeogenesis pathway that catalyze the two irreversible reactions distinguishing neoglucogenesis from glycolysis, the other one being *ppsA*, encoding phosphoenolpyruvate synthase. PpsA was also identified in our proteomic approach as increasing in the Δ*vsr217* mutant, suggesting that it could also be negatively regulated by Vsr217. However, *ppsA* mRNA was not predicted as a target by CopraRNA. Further studies will be required to determine the potential involvement of Vsr217 in *ppsA* expression.

**FIG 7:**
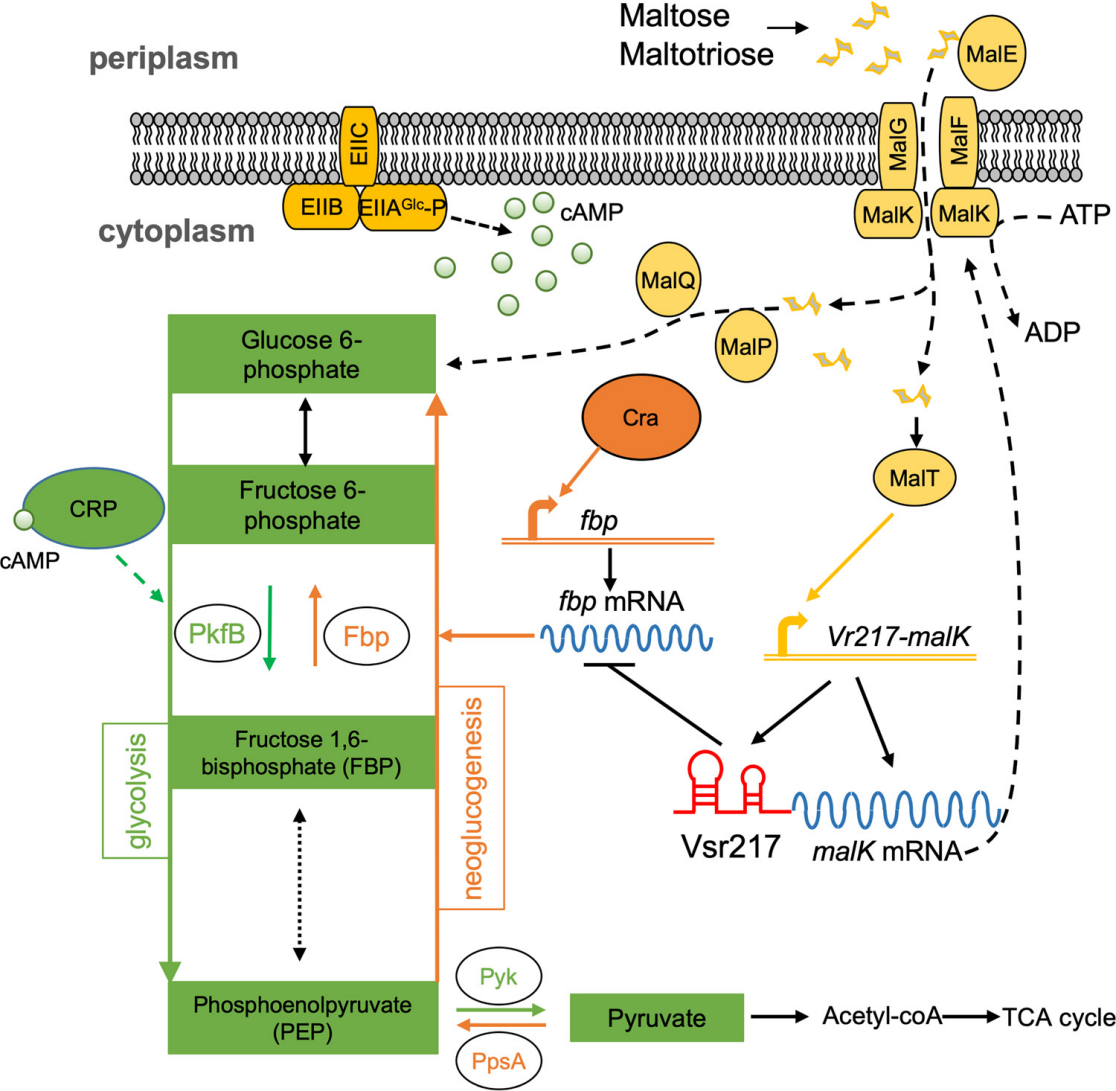
Role of Vsr217 in carbon metabolism regulation of *V. tasmaniensis*. The model is inferred from knowledge in enterobacteria and the existence of CRP (VS_2855) and Cra (VS_II1031) orthologs in *V. tasmaniensis*. In the presence of maltose or maltotriose as a carbon source instead of glucose, EIIA(glc) is phosphorylated and activates adenylate cyclase (not shown). cAMP accumulates and activates the cAMP receptor protein (CRP), which regulates positively glycolysis. On the other hand, the catabolite repressor activator Cra stimulates gluconeogenesis by activating *fbp* (and possibly *ppsA*). Maltose activates the MalT regulon including maltose transport genes such as *malK*, *malE*, *malFG* and maltose metabolic genes *malPQ*, which transform maltose into the first compound of glycolysis, glucose 6-phosphate. Vsr217 is highly expressed and represses *fbp* expression, inhibiting gluconeogenesis and favoring optimal maltose utilization. Glycolysis is in green, with only relevant steps for this study being shown. Gluconeogenesis, orange; maltose regulon, yellow; glucose PTS transporter, dark yellow; mRNAs, blue; Vsr217, red.

In E. coli, two major transcriptional regulators have been described to regulate the central carbon metabolism, CRP (catabolite repressor protein) and Cra (catabolite repressor activator, also known as FruR) (30–32). Whereas on neoglucogenic substrates such as acetate, CRP activates key enzymes of glycolysis, Cra downregulates the majority of enzymes in the glycolytic pathway and activates the two gluconeogenesis genes *fbp* and *ppsA*. The action of Cra is dominant over that of CRP (33). Both CRP and Cra have homologues in *V. tasmaniensis*, VS_2855 and VS_II1031, respectively. We propose that Vsr217 contributes to fine-tuning the production of a gluconeogenic enzyme, bringing an additional control to *Vibrio* metabolic regulations (Fig. 7).

In the presence of maltose and absence of glucose, the subunit EIIA of the glucose phosphotransferase system (PTS) transporter is phosphorylated and activates adenylate cyclase: cAMP accumulates in the cell, activating CRP, which upregulates glycolysis. Maltose is transformed into glucose-6-phosphate and enters the glycolytic pathway. However, upregulation of glycolysis is counteracted by Cra, which drives the carbon flux toward gluconeogenesis. On the other hand, induction of the maltose regulon leads to a strong increase of Vsr217 that will inhibit the expression of the gluconeogenic enzyme Fbp, thus favoring glycolysis. Hence, in oysters, which are rich in the maltose precursor glycogen, Vsr217, a member of the growing family of *trans-*acting sRNAs derived from a 5′ UTR, may contribute to the fitness of *V. tasmaniensis* by optimizing maltose utilization through inhibition of gluconeogenesis.

## MATERIALS AND METHODS

### Bacterial strains and plasmids.

Bacterial strains and plasmids used in this study are listed in Table 1. *V. tasmaniensis* LGP32 mutants were constructed by allele replacement through homologous recombination as described previously (34). The presence of the desired mutations was confirmed by sequencing. Plasmids were constructed by one-step isothermal assembly (Gibson assembly) (35). Their main features are indicated in Table 1, and details of construction are presented in Fig. S2 in the supplemental material. We also constructed a derivative of the cloning vector pXG-10 (26), deleting the fragment coding for the alpha peptide present upstream of the *gfp* gene which led to a background expression of *gfp*. The resulting plasmid, pXG-10Δalpha was used as a control plasmid to determine the basal intrinsic cellular fluorescence level. Plasmids expressing either Vsr217 or MalK alone, under the control of their native promoters P1 and P2, were constructed by deleting either coding region from a parental plasmid carrying the complete locus P1-*vsr217*-P2-*malK* (Fig. S2). Primers used for DNA amplification are listed in Table 3.

**TABLE 3:** Oligonucleotides used in this study

Name	Sequence (5′→3′)^*a*^	Target/goal
Northern blot probes		
NB-vs-MalK	CATACCTACACCACGTTTTGATGGCC	*malK*
NB-vs-Vsr217	CCCGTGATTCAATCACAGATTTAGCTCGA	Vsr217
NB-vs-Fbp	ATCTCACGGTTAACAATTTTTGCAG	*fbp*
NB-vs-tmRNA	AGCCACGCTAGCTAGTTTGGGTTTGATCT	tmRNA
RT-PCR		
Vsr217-RT-F	ACCAGCGAATGTTATGCGTT	Vsr217
MalK-RT-R	CAAGCGACGACATGCTCTAC	*malK*
5′ and 3′ RACE		
Oligo(dT)-anchor primer	GACCACGCGTATCGATGTCGACTTTTTTTTTTTTTTTT	RACE product cloning
PCR Anchor primer	GACCACGCGTATCGATGTCGAC	RACE product cloning
5′ Malk-GSP1	GAATACCATACCTACACCACGTTTT	*malK*
5′ Malk-GSP2	ACGTAATATCTTCTAGACCTGCGAT	*malK*
5′ Malk-GSP3	GAACTCACCTTCGTTGATGTCTAAG	*malK*
5′ Vsr217-GSP1	GGTGGGACTTGAATTCTTTAACCAA	Vsr217
5′ Vsr217-GSP2	CACCCGTCATAGCCAAACTG	Vsr217
5′ Vsr217-GSP3	CCAACTAAATGAACAATGCGTCAAC	Vsr217
3′ Malk-GSP1	TGCGACGATTGAAGCTGATG	*malK*
3′ Malk-GSP2	GGTATTCCTGCACACCGTTG	*malK*
3′ Vsr217-GSP1	ACCAGCGAATGTTATGCGTT	Vsr217
3′ Vsr217-GSP2	CTGCCCGCTGTTGTCTTTAT	Vsr217
*vsr217*- and *malK*-expressing plasmids		
pGEB12-F	CACTGGCCGTCGTTTTACAACGTCG	pGEB12
pGEB12-R	CAAGCTTGCATGCCTGCAGGTCGAC	pGEB12
Vsr217-malK-F	aggcatgcaagcttgCCCTTTATCTACTCCTCCCC	P1-*vsr217*-P2-*malK* plasmid
Vsr217-malK-R	aaacgacggccagtgAACAGACAAAGCGCGATC	P1-*vsr217*-P2-*malK* plasmid
del vsr217-F	TTACTCACTCAATACTTACCAATTCCTACAGTTACTGCTG	Deleting *vsr217* to get pP1-P2-MalK
del vsr217-R	GGTAAGTATTGAGTGAGTAAGAATGCAGTTTCGGTGAATT	Deleting *vsr217* to get pP1-P2-MalK
del malK-F	TCGGTTCGTCTTTTTAGAATATAACTTGCCCGTATCGAAT	Deleting *malK* to get pP1-Vsr217
del malK-R	TTCTAAAAAGACGAACCGAATTGAGTGAGTAAGAAAAAAGTGGGTGGG	Deleting *malK* to get pP1-Vsr217
Mutagenesis		
pSW7848-F	GTCTGATTCGTTACCAATTATGACAAC	Vector amplification
pSW7848-R	GAATTCGATATCAAGCTTATCGATAC	Vector amplification
Vsr217-up-F	aagcttgatatcgaattcTCTTCCCAAGACTTAGGTGGGTTAGGGACAAG	Upstream of *vsr217*
Vsr217-up-R	agtgagtaaGAATGCAGTTTCGGTGAATTCGCGTAATGG	Upstream of *vsr217*
Vsr217-down-F	ctgcattcTTACTCACTCAATACTTACCAATTCCTACAGTTACTGCTGAC	Downstream of *vsr217*
Vsr217-down-R	ttggtaacgaatcagacCCAGCAACGAAACGGTTTTGAGGATAGTGG	Downstream of *vsr217*
malT-up-F	aagcttgatatcgaattcTATTCGTAGTGAAGACCGTAGCC	Upstream of *malT*
malT-up-R	cgaatcgtCTATTCCGTGGCGTATGTTTTG	Upstream of *malT*
malT-down-F	cggaatagACGATTCGAGATTGATTCG	Downstream of *malT*
malT-down-R	ttggtaacgaatcagacTAATGCTGTAAGCGAAGG	Downstream of *malT*
polyT up-F	ataagcttgatatcgaattcAGACTTAGGTGGGTTAGG	Upstream of last 13 nt of *vsr217*
polyT up-R	gagtgagtaaGGGACTTGAATTCTTTAACC	Upstream of last 13 nt of *vsr217*
polyT down-F	ttcaagtcccTTACTCACTCAATACTTACCAATTC	Downstream of last 13 nt of *vsr217*
polyT down-R	taattggtaacgaatcagacCAATCGTCGCATCACCTG	Downstream of last 13 nt of *vsr217*
sRNA interaction system		
pZE12-F	CTAGAGGCATCAAATAAAACGAAAGGCTCA	pZE12MCS
pZE12-R	GGTACCTTTCTCCTCTTTAATGAATTCGGT	pZE12MCS
Vsr217-F	ttaaagaggagaaaggtaccCCCTTTATCTACTCCTCCC	*vsr217*
Vsr217-R	gttttatttgatgcctctagAACAGACAAAGCGCGATC	*vsr217*
pXG-10_fwd	GCTAGCAAAGGAGAAGAACTTTTC	pXG-10 (vector amplification)
pXG-10_rev	GTGCTCAGTATCTCTATCA	pXG-10 (vector amplification)
pZE-CAT	TGGGATATATCAACGGTGGT	pXG-10 (sequencing insert) (26)
JV0-155	CCGTATGTAGCATCACCTTC	pXG-10 (sequencing insert) (26)
Vibtas_FBP_UTR_F	atagagatactgagcacATTACCATTAAAAAGATCTAACCTTTAG	*fbp* 5′ UTR (−125, +3)
Vibtas_FBP_UTR_R_bis	agttcttctcctttGCTAGCCATGTTATTTCCTTAAACTTCTCTC	*fbp* 5′ UTR (−125, +3)
Ptet-R-2	GTGCTCAGTATCTCTATCACTGATA	Deleting the alpha peptide encoding fragment from pXG-10
GFP-F	GCTAGCAAAGGAGAAGAACTTTTC	Deleting the alpha peptide encoding fragment from pXG-10

a Nontarget bases complementary to the cloning vector are indicated by lowercase letters.

10.1128/mSphere.00134-21.2 FIG S2 Locus *vsr217-malK* and construction of plasmids used in this study. (Top) Genetic structure of the *vsr217-malK* locus in *V. tasmaniensis* LGP32. *vsr217*, red and capital letters; *malK* 5′ UTR (26 nt), green and lowercase letters; *malK* open reading frame (ORF) (ATG to stop codon; not shown entirely), green and capital letters; predicted −35 and −10 sequences of P1 and P2 promoters, bold and underlined letters; putative MalT box, yellow letters. The 13 nucleotides deleted in the Δ*polyT* mutant are in italics. The shown sequence was inserted in pGEB12 to generate the pP1-VSR217-P2-MalK plasmid, comprising the totality of the locus. The pP1-P2-MalK and pP1-Vsr217 plasmids were then generated from this plasmid by, deleting the *vsr217* sequence (red and capital letters) and the *malK* sequence (green), respectively, keeping the P2 promoter and the transcription terminator (highlighted by a green box). (Middle) Structure of pLac-Vsr217. The *vsr217-malK* locus including its terminator (capital letters) was inserted under the control of the pLacO promoter present on the pZE12 plasmid (26), and the *malK* gene was then deleted from the plasmid as for the pP1-Vsr217 plasmid. (Bottom) Structure of the pTet-FBP-GFP plasmid. The 5′ UTR of the *V. tasmaniensis fbp* gene (in capital letters, with ribosome binding site (RBS) and ATG in boldface font) was inserted in the pXG-10 plasmid (26) under the control of the pLtetO promoter, generating a fusion of the ATG with the 2nd codon of the *gfp* gene present on the plasmid (in green). The putative target region in *fbp* 5′ UTR is in italics. Download FIG S2, PDF file, 0.1 MB. Copyright © 2021 Luo et al. 2021 Luo et al. https://creativecommons.org/licenses/by/4.0/ This content is distributed under the terms of the Creative Commons Attribution 4.0 International license.

### Media and bacterial growth.

E. coli strains were cultured at 37°C in Luria-Bertani (LB) broth. *V. tasmaniensis* LGP32 and its derivative were grown at 20°C in Zobell medium (4 g/liter peptone, 1 g/liter yeast extract, 0.1 g/liter ferric phosphate, and 30 g/liter sea salt) or minimal medium [3 g/liter KH_2_PO_4_, 7 g/liter K_2_HPO_4_, 2 g/liter (NH4)_2_SO_4_, 0.5 × 10**^−^**3 g/liter FeSO_4_, 2 × 10**^−^**^3^ M MgSO_4_ and 30 g/liter NaCl] supplemented with 2 g/liter d-glucose or d-maltose, where indicated. Antibiotics were used as follows: chloramphenicol (Cm) at 20 μg/ml and ampicillin (Amp) at 100 μg/ml for E. coli and Cm at 2 μg/ml for LGP32. IPTG was used at 0.5 mM.

To measure growth of LGP32, bacteria (3 replicate cultures in each case) were cultured overnight in minimal medium supplemented with 2 g/liter d-glucose or d-maltose. If needed, Cm was added. Overnight cultures were diluted to an optical density at 600 nm (OD_600_) of ∼0.05 in the same medium, and growth was monitored in a 96-well plate, by measuring cell density at OD_600_ at regular time intervals in a CLARIOstar (BMG Labtech, France) microplate reader.

### RT-PCR and Northern blotting.

Overnight cultured bacteria were diluted to an OD_600_ of ∼0.05 in minimal medium, supplemented by glucose or maltose where indicated, except for the results presented in Fig. 2, where cells were grown in Zobell medium, since the *malT* mutant does not grow in minimal medium plus maltose. Cells were grown with continuous shaking at 180 rpm at 20°C. When the OD_600_ reached ∼0.4, bacteria were collected by centrifugation and resuspended in TRI reagent (Sigma) before total RNA extraction and purification, which was carried out using Direct-zol MiniPrep Plus (Zymo research). DNA was removed by TURBO DNase (Ambion) treatment. RNA was further purified by phenol-chloroform extraction and RNA precipitation. Ten micrograms (5 μg in the case shown in Fig. 4B) purified RNA was used for Northern blotting as described previously (19). Oligonucleotide probes (Table 3) complementary to the gene to be assayed were labeled at their 3′ ends using terminal transferase (Fermentas, USA) and [α-^32^P]dCTP, according to the manufacturer’s instructions. Transfer-messenger RNA (tmRNA) was used as a loading control. Membranes were scanned using an Amersham Typhoon biomolecular imager (GE Healthcare) in the phosphorimager mode. Transcript signals were quantified by ImageJ (36) and normalized to the signal of tmRNA.

For RT-PCR, 1 μg RNA was used as a template for the first-strand synthesis catalyzed by SuperScript III reverse transcriptase (Invitrogen). Amplification of the *vsr217-malK* transcript was then carried out using Dream*Taq* DNA polymerase (Thermo Fisher Scientific) and primers Vsr217-RT-F and MalK-RT-R (Table 3).

### 5′ and 3′ rapid amplification of cDNA ends.

Overnight cultures of LGP32 WT and Δ*vsr217* strains were diluted to an OD_600_ of ∼0.05 and grown in minimal medium supplemented with 2 g/liter d-maltose. Bacteria were collected by centrifugation when the OD_600_ reached ∼0.4 and were resuspended in TRI reagent followed by RNA extraction, DNase treatment, and RNA purification. 5′ and 3′ ends of Vsr217 and *malK* transcripts were determined using the 5′/3′ RACE system for rapid amplification of cDNA ends (Invitrogen). RNA primers used are listed in Table 3. In each case, the subsequent PCR products were cloned in the pJET1.2 vector (Thermo Fisher Scientific). Twenty plasmids were prepared from randomly chosen colonies, and inserts were sequenced using oligonucleotides pJET1.2-F/R (Table 3).

### RNA stability measurement.

LGP32 WT, Δ*vsr217*, and Δ*polyT* strains were grown overnight in minimal medium supplemented with 2 g/liter d-maltose at 20°C. Bacteria were diluted to an OD_600_ of ∼0.05 and grown in the same medium until an OD_600_ of ∼0.4 and rifampicin was added to a final concentration of 500 μg/ml. Bacteria were collected for RNA extraction at 0, 0.5, 1.0, 2.0, and 3.0 min after the addition of rifampicin. Northern blot analysis was performed to determine the amount of Vsr217 and *malK* transcripts.

### GFP fluorescence measurement.

To monitor green fluorescent protein (GFP) fluorescence, strains to be assayed (derived from E. coli MG1655) were grown overnight in LB supplemented with Cm and Amp. Five microliters of the overnight culture was spotted onto LB agar supplemented with Cm, Amp, and IPTG where indicated, and the plates were incubated at 37°C. Plates were then scanned with an Amersham Typhoon model 5 biomolecular imager (GE Healthcare), using the fluorescence mode (excitation [Exc], 488 nm; emission [Em], 525 nm; filter Cy2 525BP20). Signal intensity of the bacterial spots was determined using ImageJ and the formula (integrated density of the spot − [spot area × mean background signal]), where the background signal was the signal obtained for cells containing the non-GFP-expressing plasmid, to remove the intrinsic fluorescence of bacterial cells. Significance of the relative differences was determined by a Student’s unpaired *t* test, with four biological replicates.

### Proteomics.

*V. tasmaniensis* LGP32 and Δ*vsr217* strains were grown in triplicates in Zobell with glucose or maltose until an OD_600_ of ∼0.4 (12 samples in total). After centrifugation of 2 ml, cells were washed in Tris/EDTA (pH 7.5) plus 20% sucrose and then resuspended in NuPAGE LDS buffer (Thermo Fisher Scientific) to a final concentration equivalent to an OD_600_ of 20. Samples were heated at 95°C for 10 min before loading 10 μl of each sample on a 4% to 12% polyacrylamide gradient gel (Invitrogen). After a 5-min migration to let the samples enter the gels, they were stained in Coomassie blue and then destained.

For tandem mass spectrometry (MS/MS), proteins were subjected to an in-gel enzymatic digestion with trypsin. Nanoscale liquid chromatography-electrospray ionization-tandem mass spectrometry (nLC-ESI-MS-MS; Triple-TOF) was carried out (gradient of 120 min), and the peptides were identified by Mascot and Andromeda and quantified by MaxQuant. Statistical analyses were carried out using Perseus (37). *P* values were calculated using a Welch’s *t* test.

### Bioinformatic predictions.

Promoters were predicted using the BPROM software, and Rho-independent terminators were predicted using the FindTerm software (38). *Vibrio malK* upstream sequences were aligned using Muscle at the EBI (39). Putative Vsr217 targets were identified using CopraRNA, and interaction between Vsr217 and the *fbp* mRNA upstream region was predicted using IntaRNA (23).
