# Supplementary figures and images for: A Small Regulatory RNA Generated from the malK 5′ Untranslated Region Targets Gluconeogenesis in Vibrio Species
Source: mSphere. 2021 Jun 30;6(3):e00134-21. doi: 10.1128/mSphere.00134-21 (PMC8265627; doi:10.1128/mSphere.00134-21)

**A**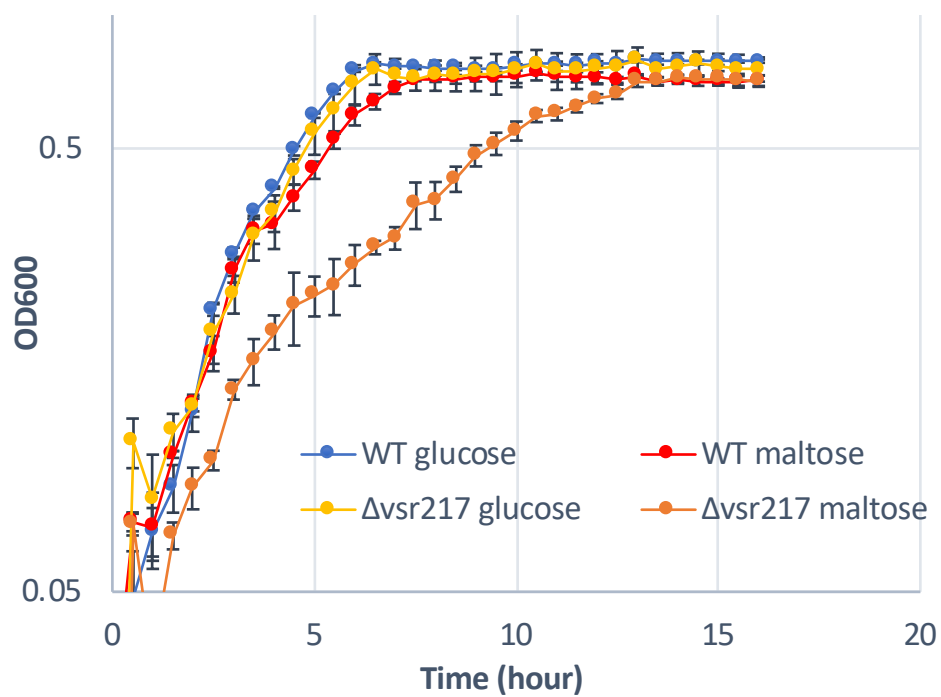**B**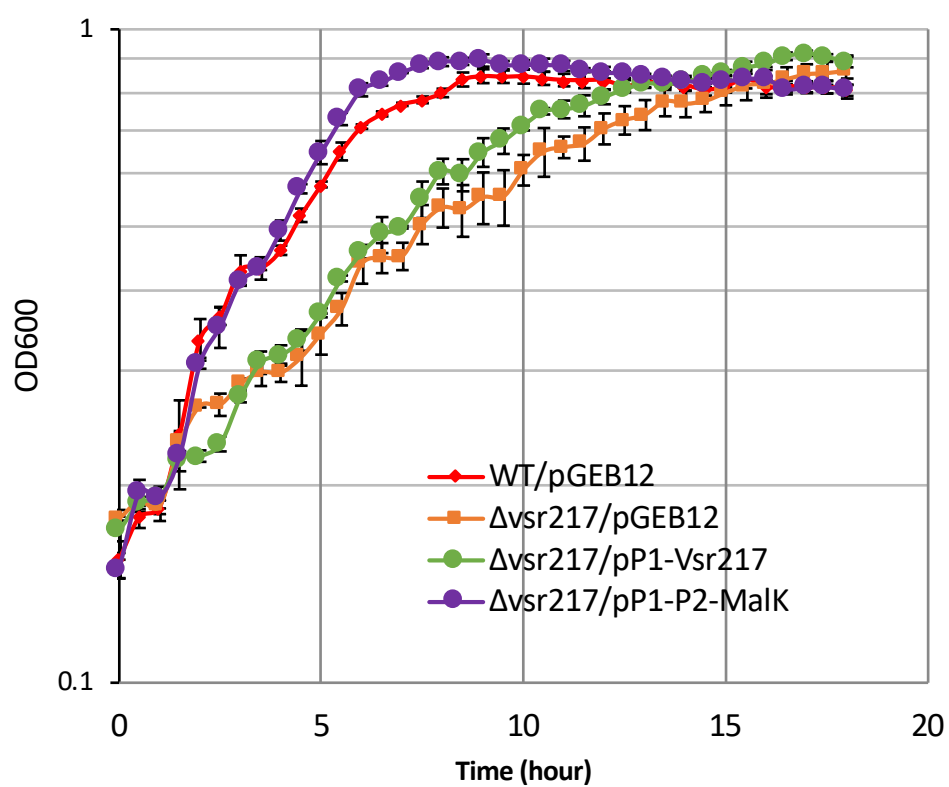

Figure S1

Supplement: FIG S1 [file msphere.00134-21-sf001.pdf]
