# Supplementary material for: A Small Regulatory RNA Generated from the malK 5′ Untranslated Region Targets Gluconeogenesis in Vibrio Species
Source: mSphere. 2021 Jun 30;6(3):e00134-21. doi: 10.1128/mSphere.00134-21 (PMC8265627; doi:10.1128/mSphere.00134-21)
Supplement: FIG S2 [file msphere.00134-21-sf002.pdf]

Locus *vsr217-malK*

P1 (MalT box)

/cccctttatctactcctcccctcctactcccccctag**ttaatt**ttctattgg*gagg**atgta***__ccat__tacgcgaattcaccg
aaactgcattc[ATCCAAGAGTGGATAGTAAGAACCCTTTACCAGCGAATGTTATGCGTTGAC
GCATTGTTCATTTAGTTGGCTTCACTGCCCGCTGTTGTCTTTATTAAGCATCGAGCTAAATC
TGTGATTGAATCACGGGGGAGATTTAGCTGGATGTGGGGGAAATAGGCACCAGTTTGGC
TATGACGGGTGGGCTTGGTTAAAGAATTCAAGTCCC*ACCCACTTTTTTC*]ttactcactcaatac

P2

ttaccaattcctacag**ttactg**ctgacccaaaaatt**ccttataat**gataagcagtaaaacttaaa[atttgatcgatc**g**
aggacaagctag**ATG**GCGA………//…….CGGCACCGAC**TAA]**tcggttcgtctttttagaatataacttgc
ccgtatcgaatccctcttaccttgtaaggggggatttctcattttctgacaaccagttttactttcactaaatagatcgcgct
ttgtctgttaa/

pLac-Vsr217

pLacO

ctcgagaattgtgagcggataacaattgacattgtgagcggataacaagatactgagcacatcagcaggacgcactgaccg
aattcattaaagaggagaaaggtaccATCCAAGAGTGGATAGTAAGAACCCTTTACCAGCGAATGTTATG
CGTTGACGCATTGTTCATTTAGTTGGCTTCACTGCCCGCTGTTGTCTTTATTAAGCATCGAGCTAA
ATCTGTGATTGAATCACGGGGGAGATTTAGCTGGATGTGGGGGAAATAGGCACCAGTTTGGCT
ATGACGGGTGGGCTTGGTTAAAGAATTCAAGTCCCACCCACTTTTTTCTTACTCACTCAATACTT

P2

ACCAATTCCTACAG**TTACTG**CTGACCCAAAAATT**CCTTATAAT**GATAAGCAGTAAAACTTAAA*[Δ
malK]*TCGGTTCGTCTTTTTAGAATATAACTTGCCCGTATCGAATCCCTCTTACCTTGTAAGGGGG
ATTTCTCATTTTCTGACAACCAGTTTTACTTTCACTAAATAGATCGCGCTTTGTCTGTTAA

pPtet-Fbp-GFP

pLTetO

acctcgagtccctatcagtgatagagattgacatccctatcagtgatagagatactgagcacATTACCATTAAAAA
GATCTAACCTTTAGAATTTAAGTAGGCGTCTAGGTGAATA*AAGCCCGAGTAAGAGGCAAATT*
*CTATAGTGCTCAAATCCCCC*TAATATTGAGAGAAGTTTA**AGGA**AATAAC**ATG**gctagcaaaggag
aagaacttttcactggagttgtcccaattcttgttgaattagatggtgatgttaatgggcacaaattttctgtcagtggaga
gggtgaaggtgatgctacatacggaaagcttacccttaa…..

Figure S2
